# Overexpression of Stathmin 1 Predicts Poor Prognosis and Promotes Cancer Cell Proliferation and Migration in Ovarian Cancer

**DOI:** 10.1155/2022/3554100

**Published:** 2022-02-09

**Authors:** Lekai Nie, Chen Zhang, Haiyun Song, Qianqian Zhao, Lei Cheng, Peihai Zhang, Xingsheng Yang

**Affiliations:** ^1^Department of Gynecology and Obstetrics, Qilu Hospital (Qingdao), Cheeloo College of Medicine, Shandong University, Qingdao, China; ^2^Department of Gynecology and Obstetrics, Qilu Hospital, Cheeloo College of Medicine, Shandong University, Jinan, China; ^3^Department of Central Laboratory and Mitochondrial Medicine Laboratory, Qilu Hospital (Qingdao), Cheeloo College of Medicine, Shandong University, Qingdao, China; ^4^Department of Pathology, Qilu Hospital (Qingdao), Cheeloo College of Medicine, Shandong University, Qingdao, China

## Abstract

**Purpose:**

The aim of this study was to investigate the expression of stathmin 1 (STMN1) in ovarian cancer and its effect on prognosis. The effect and mechanism of STMN1 on the proliferation and migration of ovarian cancer cells were also investigated.

**Methods:**

Expression of STMN1 was measured by immunohistochemical staining in ovarian cancer tissues. The effects of STMN1 on the proliferation and migration capacity of ovarian cancer were evaluated using Cell Counting Kit-8 (CCK-8) assays, colony formation assays, immunofluorescence staining, wound healing assays, and Transwell assays. Transcription factors were predicted by bioinformatic analysis of TCGA database.

**Results:**

STMN1 was upregulated in ovarian cancer tissues as compared to paracancerous tissues and associated with shorter overall survival. STMN1 expression significantly correlated with FIGO staging and tumor differentiation (*P* < 0.05). Furthermore, STMN1 promoted proliferation and migration in ovarian cancer cell lines. Bioinformatic analysis revealed that STMN1 was potentially regulated by E2F transcription factors. Then, we found that E2F1 regulated the expression of STMN1 and affected proliferation.

**Conclusion:**

STMN1 is overexpressed in ovarian cancer, and its high expression suggests a poor prognosis. STMN1 promotes the proliferation and migration of ovarian cancer and is regulated by E2F1. Thus, STMN1 may serve as a negative prognostic factor and possible target for the treatment of ovarian cancer patients.

## 1. Introduction

Ovarian cancer is one of the most common malignant tumors in the female reproductive organs, with its mortality rate ranking number one. The 5-year survival rate of ovarian cancer patients was 75-92% if diagnosed at localized and regional stages, while only 29% of those diagnosed at an advanced-stage survived. Unfortunately, 59% of patients are diagnosed at advanced stages [1]. There was no effective standard screening strategy for the early detection of ovarian cancer until now [2]. A recent report from the United Kingdom Collaborative Study of Ovarian Cancer Screening (UKCTOCS) showed that annual multimodal screening including biomarker CA125 and transvaginal ultrasound scans could reduce the incidence of advanced-stage ovarian cancer compared with no screening, even though the screening did not significantly reduce ovarian and tubal cancer deaths in the general papulation [3]. At present, cytoreductive surgery, chemotherapy, and maintenance therapy are the main treatment strategies for ovarian cancer [4]. Targeted therapy and individualized treatment are the future of cancer therapy. Therefore, it is necessary to explore the molecular mechanism underlying the occurrence and development of ovarian cancer.

Stathmin 1 (STMN1), also known as Op18, p18, oncoprotein 18, and metablastin, is a member of the stathmin family, which is associated with microtubule destabilization and plays an important role in the construction and function of the mitotic spindle [5]. STMN1 has been shown to be involved in the development and progression of many malignant tumors, and the overexpression of STMN1 promotes their metastasis and growth, which is associated with poor prognosis [6, 7]. However, there are few studies investigating the molecular mechanism of STMN1 in cancers. In anaplastic thyroid carcinoma, HN1 could promote tumor growth and metastasis by interacting with STMN1 [8]. In hepatocellular carcinoma, STMN1 could promote tumor progression by interacting with YAP1 [9] and triggering the MET pathway [10]. In non-small-cell lung cancer, STMN1 could increase radioresistance by enhancing autophagy [11]. In cholangiocarcinoma, STMN1 could regulate p27 expression, resulting in poor clinical prognosis [12].

To date, there have been several studies on STMN1 in ovarian cancer. Immunohistochemistry showed an apparent overexpression of STMN1 in ovarian cancer [13]. Overexpression of STMN1 is associated with paclitaxel resistance and survival in ovarian cancer [14, 15]. STMN1 regulates mutant p53 stability, transcriptional activity, and hypoxia-inducible factor-1*α* expression in ovarian cancer [16, 17]. Meanwhile, STMN1 has also been shown to be regulated by miR-193b [18], miR-31 [19], and SIVA 1 [20] in ovarian cancer. These previous studies suggested that STMN1 may function as an oncogene and may be a potential target for the treatment of ovarian cancer. However, the molecular mechanisms of STMN1 in ovarian cancer remain largely unknown. To date, it has not been confirmed whether STMN1 can promote the proliferation and migration of ovarian cancer cells in vitro. In addition, there are no studies on the relationship between STMN1 expression and clinical factors in ovarian cancer.

In our study, we aimed to investigate STMN1 expression in ovarian cancer and correlated its expression with clinical factors and outcomes. Moreover, we found that STMN1 could promote proliferation and migration in ovarian cancer cell lines. Furthermore, we demonstrated that STMN1 was regulated by E2F1, which might be an important mechanism through which STMN1 promotes proliferation in ovarian cancer cells. These findings indicated that STMN1 could be a prognostic indicator and promising therapeutic target in ovarian cancer.

## 2. Materials and Methods

### 2.1. Patients and Specimens

Fifty-five ovarian cancer samples and 10 paracancerous samples were collected between 2013 and 2016 at Qilu Hospital (Qingdao), Cheeloo College of Medicine, Shandong University, Qingdao, China. Diagnoses were confirmed using light microscopy and immunohistochemistry. Patients with other malignant tumors were excluded. Follow-up information was obtained from the outpatient records, telephone, and WeChat. Overall survival (OS) was calculated from the beginning of the treatment to the date of death or the last follow-up consultation. The study was approved by the Research Ethics Committee of Qilu Hospital (Qingdao), Cheeloo College of Medicine, Shandong University. All samples were used in accordance with institutional guidelines and the Helsinki Declaration. All samples were used with the consent of the patients.

### 2.2. Immunohistochemical Staining and Evaluation

Tumor specimens were obtained by surgical excision or biopsy. Each 5 *μ*m thick tissue section was deparaffinized, and endogenous peroxidase was blocked. Nonspecific binding sites were then blocked with goat serum. Subsequently, the slides were incubated with anti-STMN1 antibody (1 : 2000; Abcam, Cambridge, UK) at 4°C. The slides were developed using the diaminobenzidine (DAB) method and then counterstained with hematoxylin.

STMN1 protein expression was semiquantitated based on immunohistochemistry by scoring the proportion of positive cells on a 4-point scale (0, 0%; 1, 1-10%; 2, 11-50%; 3, 51-100%) and scoring the staining intensity on a 4-point scale (0, no expression; 1, weak positive expression; 2, positive expression; 3, strong positive expression). The product of the two scores determined the final staining result. All the samples were scored as 0, 1, 2, 3, 4, 6, or 9. Samples with scores of 0-4 were considered low expression, and samples with scores of 6-9 were considered high expression [21]. Evaluation of the stained sections was performed by two pathologists.

### 2.3. Cell Culture

The ovarian cancer SKOV3 cell line was purchased from Shanghai Zhongqiao Xinzhou Biotechnology Co. Ltd. and cultured with RPMI-1640 supplemented with 10% fetal bovine serum (FBS) at 37°C in 5% CO_2_. The ovarian cancer A2780 cell line was purchased from Shanghai Fuheng Biotechnology Co. Ltd. and cultured with DMEM supplemented with 10% FBS at 37°C in 5% CO_2_. RPMI-1640, DMEM, and FBS were purchased from Procell (Wuhan, China).

### 2.4. Transfection of siRNA Interference and Overexpression Plasmids

Cells at 70-90% confluency were transfected with a small interfering RNA (siRNA) or overexpression plasmid using LipoHigh transfection reagent (Sangon Biotech, Shanghai, China). The STMN1 overexpression plasmid pcDNA3.1-STMN1 and the control pcDNA3.1 were purchased from Vigene Biosciences (Jinan, China). siRNAs against STMN1 and E2F1 and control siRNA were purchased from GenePharma (Shanghai, China). The sequence of STMN1 siRNA was 5′-CUGGAACGUUUGCGAGAGA-3′. The sequence of E2F1 siRNA was 5′-GGACCUGGAAACUGACCAU-3′.

### 2.5. RNA Preparation, Complementary DNA Synthesis, and Quantitative Real-Time PCR

Total RNA was isolated using TRIzol reagent (Takara, Beijing, China) according to the manufacturer's instructions. One microgram of total RNA was used to reverse transcribe complementary DNA (cDNA) with the TB Green® Premix Ex Taq™ II system (Takara, Beijing, China). Real-time PCR (PrimeScript™ RT Reagent Kit, Takara, Beijing, China) analysis was performed using a StepOnePlus Real-Time PCR System according to the manufacturer's instructions. Primer sequences were as follows: for STMN1, 5′-AAGAGAACCGAGAGGCACAAATGG-3′ (forward), 5′-GGCAAAGGGCAGGAACAGAGTG-3′ (reverse); for E2F1 5′-CTGTGCCCTGAGGAGACCGTAG-3′ (forward), 5′-GAGATGATGGTGGTGGTGACACTATG-3′ (reverse). The data were analyzed by the 2^−ΔΔCt^ method, and *β*-actin was used as the reference gene.

### 2.6. Western Blot

Total cell protein extracts were prepared and separated by 12.5% SDS-PAGE and transferred to polyvinylidene fluoride (PVDF) membranes. The PVDF membranes were incubated with the following antibodies at 4°C overnight: rabbit antibodies to STMN1 (1 : 2000; Abcam, Cambridge, UK); rabbit antibodies to E-cadherin, vimentin, N-cadherin, and PCNA (1 : 1000; CST, Danvers, USA); and rabbit antibodies to E2F1 (1 : 1000; ABclonal, Wuhan, China). Immunoreactive proteins were visualized by enhanced chemiluminescence (Epizyme, Shanghai, China). Quantifications were carried out using ImageJ software and normalized to *β*-actin levels.

### 2.7. Cell Proliferation Assay

Cell proliferation was evaluated through a Cell Counting Kit-8 (CCK-8) assay. Approximately 2.0 × 10^3^ cells transfected with siRNA or plasmid were plated in 96-well plates in 100 *μ*L of medium and cultured at 37°C. Cell viability was detected at 24, 48, 72, 96, and 120 h after plating. Ten microliters of CCK-8 reagent was added to the 96-well plates and incubated at 37°C for 2 hours. Absorbance at 450 nm was measured by a microplate reader (Thermo Fisher, USA).

### 2.8. Colony Formation

Cells transfected with siRNA or plasmid were plated in 6-well plates at 1.0 × 10^3^ cells per well and cultured at 37°C for 2 weeks. For all experiments, cells were fixed with 4% paraformaldehyde (Absin, Shanghai, China) for 20 minutes at room temperature. Then, the cells were stained with crystal violet stain (Solarbio, Shanghai, China) for approximately 10 min. Cell clones were counted under a microscope, and clones with >50 cells were considered positive.

### 2.9. Wound Healing Assay

Cells transfected with siRNA or plasmid were cultured to create a confluent monolayer. Ten-microliter pipette tips were used to create a constant-diameter “scratch,” and wells were washed with PBS. Images of the wounds were captured at 0, 24, and 48 h with an inverted phase contrast microscope. The distance of cell migration was measured using ImageJ software.

### 2.10. Transwell Assay

Briefly, 5.0 × 10^4^ cells transfected with siRNA or plasmid were plated in a Transwell insert (8 *μ*m pore; Jet Biofil, Guangzhou, China) using medium, DMEM or RPMI-1640, containing 1% FBS. The lower chambers were filled with medium containing 10% FBS. Invaded cells were fixed and stained with 4% paraformaldehyde (Absin, Shanghai, China) and crystal violet after 24 h. Four random views were collected under a microscope and then quantified.

### 2.11. Statistical Analysis

Statistical analyses were performed using the *t*-test for continuous variables and the chi-square test for categorical variables with GraphPad Prism 5 (GraphPad Software, Inc., San. Diego, CA, USA). Overall survival was evaluated using Kaplan-Meier survival curves and compared by the log-rank test. *P* < 0.05 was considered statistically significant.

## 3. Results

### 3.1. Clinical Characteristics

The patient characteristics are shown in Table 1. The mean age of the 55 patients with ovarian cancer was 53.31 years (range 25 to 81). In postoperative pathology, 40 cases were serous cystadenocarcinoma, 7 cases were mucinous cystadenocarcinoma, 6 cases were clear cell carcinoma, and 2 cases were endometrioid carcinoma (Table 1). Eighteen cases were at stages I-II, and 37 patients were at stages III-IV. Eleven (20.00%) cases were differentiated well (G1), and 40 (72.73%) cases were differentiated moderately and poorly (G2-G3). The median OS was 46.5 months.

### 3.2. STMN1 Expression Was Upregulated in Ovarian Cancer and an Indicator of Poor Prognosis

**Figure 1:**
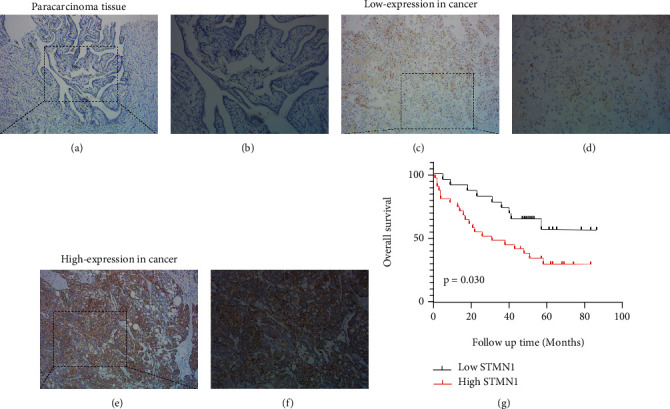
Immunohistochemical analysis of STMN1 expression in representative ovarian cancer tissue and paracarcinoma tissue samples. (a, b) Low STMN1 expression in the paracarcinoma tissue (magnification, (a) ×100; (b) ×200). (c, d) Low STMN1 expression in ovarian cancer tissue (magnification, (c) ×100; (d) ×200). (e, f) High STMN1 expression in ovarian cancer tissue (magnification, (e) ×100; (f) ×200). (g) Kaplan-Meier survival curves of ovarian cancer patients expressing high or low STMN1.

To measure STMN1 expression in ovarian cancer tissue, immunohistochemical staining was performed in ovarian cancer tissues and paracancerous tissues. We found that STMN1 expression was upregulated in ovarian cancer tissues compared with paracancerous tissues (Figures 1(a)–1(f)). The relationships between STMN1 expression and the clinicopathological characteristics of ovarian cancer patients are shown in Table 1. We found that the STMN1 expression was significantly correlated with FIGO staging and tumor differentiation (*P* < 0.05). There were no correlations between STMN1 expression and age, pathological classification, tumor size, lymph node metastasis, or CA125 level. Moreover, patients with high STMN1 expression suffered from shorter PFS and OS than those with low STMN1 expression (Figure 1(g)). Therefore, STMN1 expression may be an indicator of poor prognosis in ovarian cancer patients.

### 3.3. STMN1 Promoted the Growth and Proliferation of Ovarian Cancer Cells

**Figure 2:**
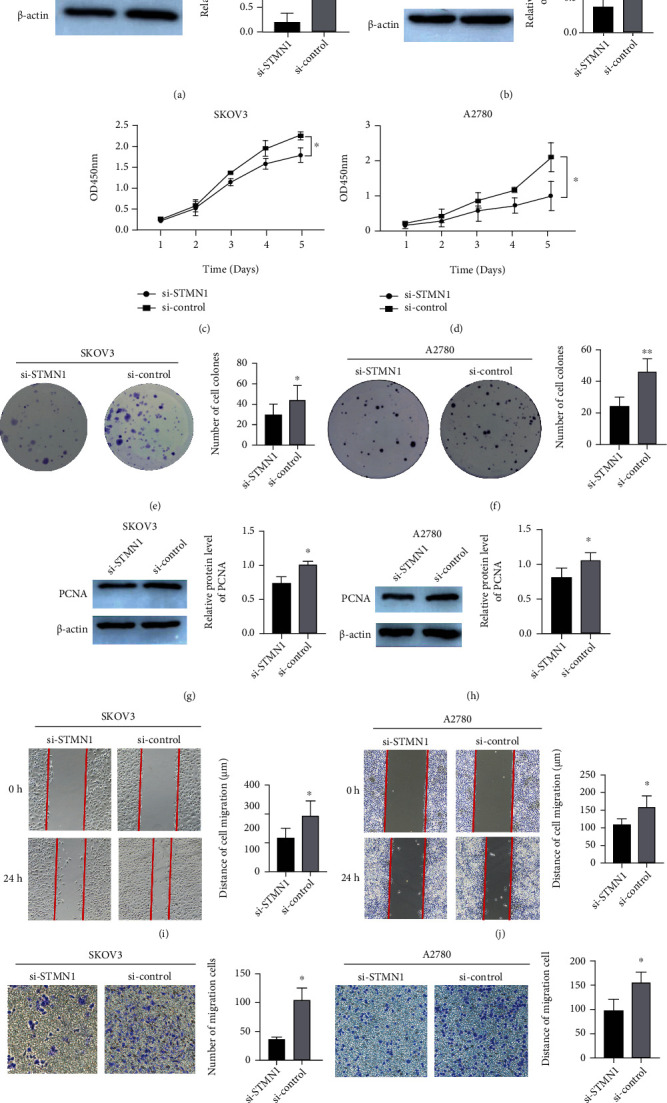
STMN1 knockdown attenuates the proliferation and migration of ovarian cancer cells. (a, b) STMN1 knockdown efficiency was confirmed by Western blot in SKOV3 and A2780 cells. (c–f) The effect of STMN1 knockdown on cell proliferation was evaluated by both the CCK-8 assay and the colony formation assay. (g, h) The protein levels of PCNA in si-STMN1 and si-control cells by Western blot. (i–l) The effect of STMN1 knockdown on cell migration was evaluated by both wound healing and Transwell assays. ^∗∗^*P* < 0.01 compared to control. ^∗∗∗^*P* < 0.001 compared to control.

**Figure 3:**
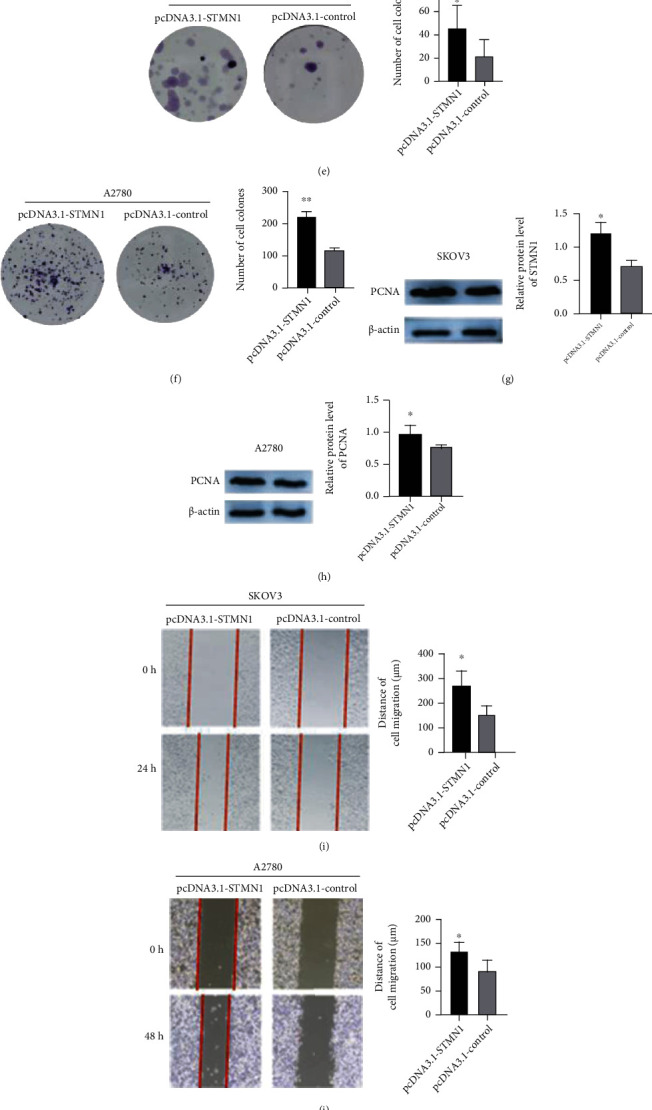
Overexpression of STMN1 enhanced the proliferation and migration of ovarian cancer cells. (a, b) STMN1 overexpression plasmid was transfected into SKOV3 and A2780 cells, and the protein level was measured by Western blot. (c–f) The effect of STMN1 upregulation on cell proliferation was evaluated by both the CCK-8 assay and the colony formation assay. (g, h) The effects of overexpression of STMN1 on the protein levels of PCNA were measured in both cell lines by Western blot. (i–l) The effect of STMN1 upregulation on cell migration was evaluated by both wound healing and Transwell assay. ^∗∗^*P* < 0.01 compared to control. ^∗∗∗^*P* < 0.001 compared to control.

To investigate the function of STMN1 in ovarian cancer, we transfected the siRNA or overexpression plasmid into ovarian cancer cells and measured cellular functions. Total RNA was isolated, and quantitative real-time PCR was performed to detect STMN1 mRNA levels while siRNAs were transfected to determine the most effective siRNA (Supplementary Figure1 (A, B)). WB was performed to detect STMN1 expression to confirm the successful transfection (Figures 2(a), 2(b), 3(a), and 3(b)). The CCK-8 assay and colony formation assay showed that STMN1 downregulation resulted in significantly reduced cell growth and fewer colonies in SKOV3 and A2780 cells than the control (Figures 2(c)–2(f)). The PCNA protein level was significantly decreased (Figures 2(g) and 2(h)). Moreover, upregulated STMN1 expression resulted in faster cell growth and increased colony formations and PCNA protein level (Figures 3(c)–3(h)).

### 3.4. STMN1 Promoted the Migration of Ovarian Cancer Cells

We performed wound healing and Transwell assays to detect the effects of STMN1 on ovarian cancer cell migration. The wound healing assay and Transwell assay demonstrated that subsequent to STMN1 downregulation, the migration distance and rate were significantly decreased compared with those of the control (Figures 2(i)–2(l)), while upregulated STMN1 expression had the opposite effects (Figures 3(i)–3(l)).

### 3.5. Bioinformatic Analysis Revealed That STMN1 Was Potentially Regulated by E2F Transcription Factors

**Figure 4:**
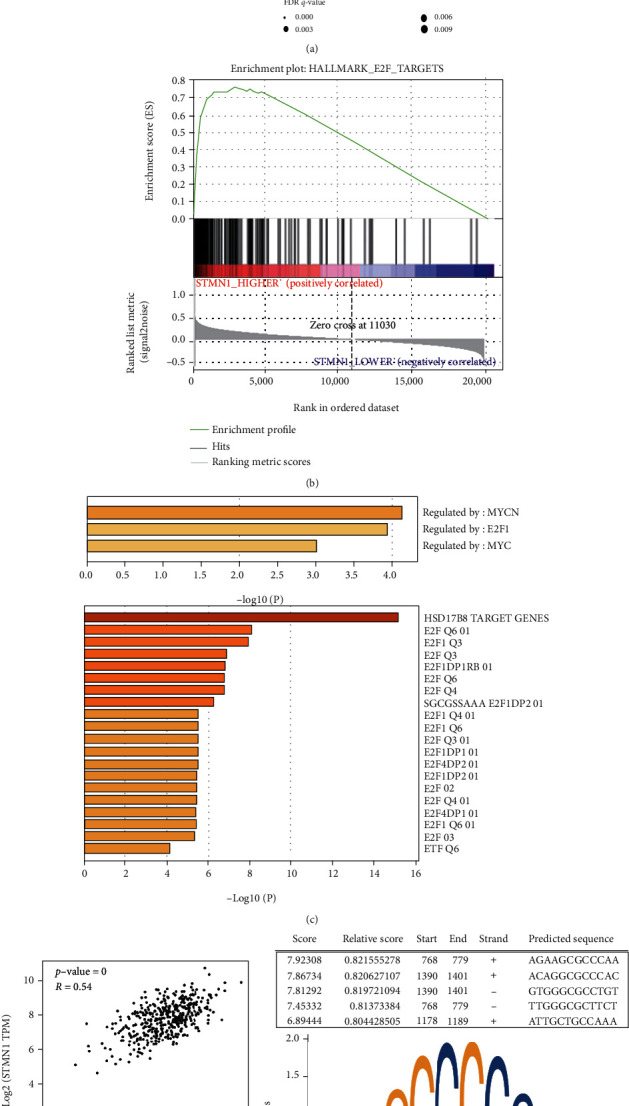
Bioinformatic analysis revealed that STMN1 was potentially regulated by E2F transcription factors. (a) A bubble chart showing the pathways of gene set enrichment analysis (GSEA). (b) GSEA showed that the gene sets associated with E2F targets were enriched in the OA samples with higher STMN1 expression. (c) Metascape analysis showed that the gene sets positively correlated with STMN1 were predicted to be regulated by E2F transcription factors in the ovarian cancer samples. (d) The expression level of STMN1 was found to be positively associated with E2F1 expression in ovarian cancer. (e) JASPAR predicted the STMN1 promoter sequence targeted by E2F1.

To determine the molecular mechanism of STMN1 in ovarian cancer, we performed gene set enrichment analysis (GSEA) to study mRNA sequencing data from 308 ovarian cancer patients in TCGA database (TCGA.OV.sampleMap/HiSeqV2_PANCAN). The group cutoff score for high or low STMN1 expression was set as the median. Pathways related to cell proliferation and cytokinesis were enriched (Figure 4(a)). Interestingly, E2F transcription factors, which play a crucial role in the control of the cell cycle, were predicted to be upregulated and target the genes associated with higher STMN1 expression (Figures 4(a) and 4(b)). To further prove the potential of E2F transcription factors in STMN1 regulation, we analyzed the top 50 genes differentially expressed in correlation with STMN1 by GEPIA in Metascape (https://metascape.org/gp/index.html#/main/step1) (Supplementary Table 1). Clearly, the E2F transcription factors were identified as possible transcriptional regulators (Figure 4(c)). In addition, E2F1, as the main member of the E2F family, positively correlated with the expression levels of STMN1 by correlation analysis using the public database GEPIA (Figure 4(d)). To identify promoter regulatory sequences, we performed motif analysis of the genomic regions to which E2F1 bound in the promoters of STMN1 by JASPAR, a database of transcription factor binding profiles (http://jaspar.genereg.net/). Five potential elements were identified with high scores, suggesting the possibility of E2F1-dependent gene regulation of STMN1 (Figure 4(e)).

### 3.6. STMN1 Was Regulated by E2F1, and E2F1 Promoted Proliferation in Ovarian Cancer Cells

**Figure 5:**
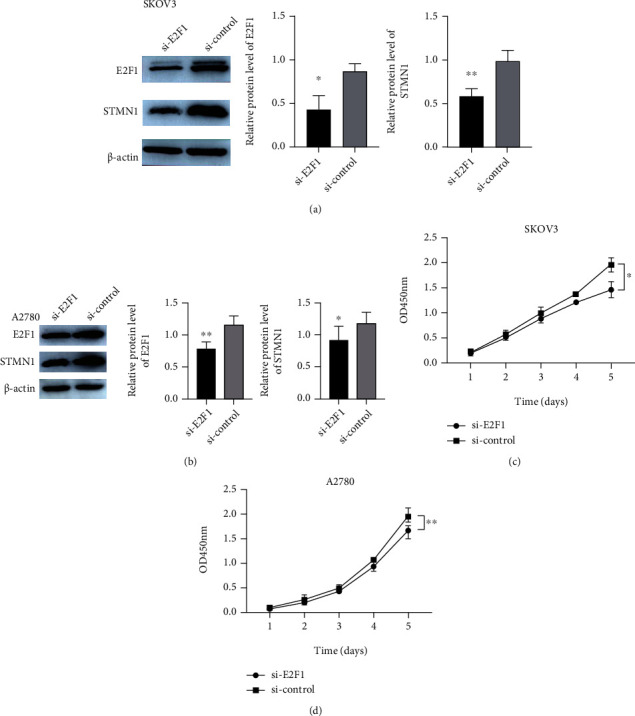
STMN1 was regulated by E2F1, and E2F1 promoted the proliferation of ovarian cancer cells. (a, b) E2F1 knockdown efficiency was confirmed by Western blot in SKOV3 and A2780 cells, and this knockdown had a significant effect on the protein expression levels of STMN1. (c, d) The effect of E2F1 knockdown on cell proliferation was evaluated by the CCK-8 assay.

We verified the association between STMN1 and E2F1 by Western blot. The expression of E2F1 significantly decreased after the si-E2F1 interference transfection. Meanwhile, the STMN1 protein level was significantly decreased in SKOV3 and A2780 cell lines (Figures 5(a) and 5(b)). The possible correlation between STMN1 and E2F1 was confirmed. The CCK-8 assay showed that cell proliferation was reduced when E2F1 was downregulated in both cell lines compared with the control (Figures 5(c) and 5(d)).

## 4. Discussion

In recent years, advances in ovarian cancer treatment have been made in surgical therapy, chemotherapy, antiangiogenic agents, and poly ADP-ribose polymerase (PARP) inhibitors (PARPis). Primary debulking surgery (PDS) followed by chemotherapy or neoadjuvant chemotherapy (NACT) followed by interval debulking surgery (IDS) is the indispensable part of ovarian cancer treatment. No residual tumor (R0) after surgery is the most important factor for survival [22]. Currently, platinum in combination with paclitaxel is the standard first-line chemotherapy regimen for ovarian cancer [4]. Paclitaxel acts by stabilizing the microtubule polymers to block disassembly of microtubules [23], which has an opposite effect to STMN1, as it is related to microtubule destabilization [5]. Thus, high STMN1 expression is associated with paclitaxel resistance and poor prognosis in cancers [24–26]. Two randomized trials, GOG218 [27] and ICON7 [28], showed the effect of antiangiogenic agent bevacizumab administration, which had a significantly increased PFS, but not OS. It is evident that the intensive investigation and application of PARPis have promoted treatment progress. PARPis have been shown to improve progression-free survival (PFS) for high-grade serous ovarian cancer patients, particularly for those with deleterious BRCA mutations [29]. One clinical trial (SOLO2/ENGOT-Ov21) demonstrated that olaparib provided a median OS benefit of 12.9 months compared with placebo in patients with platinum-sensitive, relapsed ovarian cancer and a BRCA1/2 mutation [30]. Therefore, efficiently applying the knowledge gained from molecular studies will guide the decision-making process and be the trend in ovarian cancer treatment. The discovery of new biomarkers for both prognosis prediction and molecular targeted therapy remains vital.

In this study, we found that STMN1 expression was significantly elevated in ovarian cancer tissues. The level of STMN1 expression was closely associated with pathological differentiation and clinical stage in patients with ovarian cancer. The higher expression level of STMN1 also correlated with shorter overall survival. Similarly, the high expression of STMN1 was confirmed in other cancers, including colorectal cancer [31], liver cancer [9], gastric cancer [24], and breast cancer [32]. These findings suggested that STMN1 may play an important role in the development of various cancers and serve as a predictor for unfavorable outcomes in cancer patients.

The effect of STMN1 on ovarian cancer proliferation and migration was also confirmed through in vitro experiments. Downregulating STMN1 expression inhibited cell proliferation and migration of ovarian cancer cell lines, and upregulating STMN1 expression had the opposite effect. It has been indicated that the expression of STMN1 affected microtubule destabilization and mitosis. Paclitaxel, currently used as a chemotherapeutic drug in ovarian cancer, induces stabilization of microtubules and arrests the cell cycle, which result in the death of cancer cells [33]. Due to the opposing mechanism, the overexpression of STMN1 resulted in the chemoresistance of paclitaxel in cancers [24, 34, 35], and knockdown of STMN1 could enhance the chemosensitivity of cancer cells [26, 36].

By correlation analysis from the public database GEPIA, we found that STMN1 was potentially regulated by the E2F transcription factors. E2F1 is a member of the E2F family that plays a crucial role as transcription factors, possessing several evolutionally conserved domains including a DNA-binding domain to regulate the cell proliferation [37]. Moreover, E2F1 plays a role in regulating cancer characteristics such as tumor proliferation, apoptosis, metastasis, and metabolism processes [38, 39]. The transactivation capacity of E2F1 is regulated by pRb, as pRb binds and inactivates the DNA binding and transactivating functions of E2F1 [40]. Previous studies have identified that E2F1 can induce STMN1 expression through promoting the transcription. In hepatocellular carcinoma (HCC) and nasopharyngeal carcinoma, the expression of E2F1 and STMN1 was correlated with each other both at mRNA and protein levels [41, 42]. Meanwhile, the interaction of E2F1 and STMN1 was ascertained by ChIP assay and dual luciferase assay in HCC and liver cancer [41, 43]. In our study, we disclosed the correlation of E2F1 and STMN1 at protein level and inferred the binding domain by bioinformatic analysis, indicating that STMN1 was regulated by E2F1 at transcriptional level in OvCas.

In addition, some mechanisms by which STMN1 is involved in cell migration have been revealed. STMN1 knockdown inhibited the migration and invasion of gallbladder cancer cells induced by the MUC16 C-terminal polypeptide [44]. The interaction between STMN1 and GRP78 correlated with breast cancer cell migration ability [32]. STMN1 plays a role in cell migration through the EMT process in hypopharyngeal squamous cell carcinoma and gastric cancer [45, 46]. Under glucose deficiency conditions, the hKIS/p27/E2F1 axis regulates STMN1 expression and gallbladder carcinoma cell migration and invasion [47].

However, there are also some limitations about this study. The main limitation of this study was that the sample size was too small. Although we found that STMN1 expression was an indicator of poor prognosis in these patients, it is necessary to further enlarge the sample size to make the conclusions more convincing.

## 5. Conclusions

In summary, these results suggest that STMN1 is overexpressed in ovarian cancer tissues and that high expression indicates a poor prognosis. STMN1 plays a crucial role in the proliferation and migration of ovarian cancer cells. STMN1 could interact with E2F1 to affect the tumor process. STMN1 may be a novel indicator for the adverse prognosis of ovarian cancer patients and a promising target for the development of new treatments.

## Figures and Tables

**Table 1:** The correlation between STMN1 expression and clinical characteristics in patients with ovarian cancer.

Clinical parameters	Low STMN1 expression (*n* = 23)	High STMN1 expression (*n* = 32)	*P* value
Age (years)			
≤60	19	20	0.105
>60	4	12	
Pathological classification			
Serous cystadenocarcinoma	14	26	0.389
Mucinous cystadenocarcinoma	4	3	
Clear cell carcinoma	4	2	
Endometrioid carcinoma	1	1	
FIGO staging			
I-II	12	6	0.009^∗∗^
III-IV	11	26	
Tumor differentiation			
G1	9	2	0.001^∗∗^
G2-G3	10	28	
Tumor size			
≤5 cm	4	9	0.355
>5 cm	19	23	
Lymph node metastasis			
Yes	3	12	0.094
No	12	14	
CA125 level			
≤500 U/mL	17	19	0.263
>500 U/mL	6	13	

Notes: ^∗∗^*P* < 0.01. Abbreviations: FIGO: International Federation of Gynecology and Obstetrics; CA125: carbohydrate antigen 125.

## Data Availability

The data used to support the findings of this study are included within the article and the supplementary information files.

## Supplementary Materials

Supplementary Materials Supplementary Figure 1: STMN1 was knocked down by siRNAs. (A, B) qRT-PCR was performed to measure STMN1 mRNA levels in SKOV3 and A2780 cells transfected with siRNAs. The sequences of STMN1 siRNA were as follows: S1, 5′-GCACGAGAAAGAAGUGCUU-3′; S2, 5′-CUGGAACGUUUGCGAGAGA-3′; and S3, 5′-GAACAACAACUUCAGUAAA-3′.

## References

[1] Siegel R. L., Miller K. D., Jemal A. (2020). Cancer statistics, 2020. *CA: A Cancer Journal for Clinicians*.

[2] Matulonis U. A., Sood A. K., Fallowfield L., Howitt B. E., Sehouli J., Karlan B. Y. (2016). Ovarian cancer. *Nature Reviews Disease Primers*.

[3] Menon U., Gentry-Maharaj A., Burnell M. (2021). Ovarian cancer population screening and mortality after long-term follow-up in the UK Collaborative Trial of Ovarian Cancer Screening (UKCTOCS): a randomised controlled trial. *The Lancet*.

[4] Kuroki L., Guntupalli S. R. (2020). Treatment of epithelial ovarian cancer. *BMJ*.

[5] Rana S., Maples P. B., Senzer N., Nemunaitis J. (2008). Stathmin 1: a novel therapeutic target for anticancer activity. *Expert Review of Anticancer Therapy*.

[6] Zhang D., Dai L., Yang Z., Wang X., LanNing Y. (2019). Association of STMN1 with survival in solid tumors: a systematic review and meta-analysis. *The International Journal of Biological Markers*.

[7] Mao Q., Chen Z., Wang K., Xu R., Lu H., He X. (2018). Prognostic role of high stathmin 1 expression in patients with solid tumors: evidence from a meta-analysis. *Cellular Physiology and Biochemistry*.

[8] Pan Z., Fang Q., Li L. (2021). HN1 promotes tumor growth and metastasis of anaplastic thyroid carcinoma by interacting with STMN1. *Cancer Letters*.

[9] Liu Y. P., Pan L. L., Kong C. C. (2020). Stathmin 1 promotes the progression of liver cancer through interacting with YAP1. *European Review for Medical and Pharmacological Sciences*.

[10] Zhang R., Gao X., Zuo J. (2020). STMN1 upregulation mediates hepatocellular carcinoma and hepatic stellate cell crosstalk to aggravate cancer by triggering the MET pathway. *Cancer Science*.

[11] Zhang X., Ji J., Yang Y., Zhang J., Shen L. (2016). Stathmin1 increases radioresistance by enhancing autophagy in non-small-cell lung cancer cells. *Oncotargets and Therapy*.

[12] Watanabe A., Suzuki H., Yokobori T. (2014). Stathmin1 regulates p27 expression, proliferation and drug resistance, resulting in poor clinical prognosis in cholangiocarcinoma. *Cancer Science*.

[13] Price D. K., Ball J. R., Bahrani-Mostafavi Z. (2000). The phosphoprotein Op18/stathmin is differentially expressed in ovarian cancer. *Cancer Investigation*.

[14] Su D., Smith S. M., Preti M. (2009). Stathmin and tubulin expression and survival of ovarian cancer patients receiving platinum treatment with and without paclitaxel. *Cancer*.

[15] Ying L., Su D., Zhu J., Ma S., Katsaros D., Yu H. (2013). Genotyping of stathmin and its association with clinical factors and survival in patients with ovarian cancer. *Oncology Letters*.

[16] Sonego M., Schiappacassi M., Lovisa S. (2013). Stathmin regulates mutant p53 stability and transcriptional activity in ovarian cancer. *EMBO Molecular Medicine*.

[17] Tamura K., Yoshie M., Miyajima E., Kano M., Tachikawa E. (2013). Stathmin regulates hypoxia-inducible factor-1alpha expression through the mammalian target of rapamycin pathway in ovarian clear cell adenocarcinoma. *ISRN Pharmacology*.

[18] Li H., Xu Y., Zhao D. (2020). MicroRNA-193b regulates human ovarian cancer cell growth via targeting STMN1. *Experimental and Therapeutic Medicine*.

[19] Hassan M. K., Watari H., Mitamura T. (2015). P18/Stathmin1 is regulated by miR-31 in ovarian cancer in response to taxane. *Oncoscience*.

[20] Ma Y., Liu T., Song X. (2017). Siva 1 inhibits proliferation, migration and invasion by phosphorylating stathmin in ovarian cancer cells. *Oncology Letters*.

[21] Watanabe A., Araki K., Yokobori T. (2017). Stathmin 1 promotes the proliferation and malignant transformation of pancreatic intraductal papillary mucinous neoplasms. *Oncology Letters*.

[22] Lheureux S., Gourley C., Vergote I., Oza A. M. (2019). Epithelial ovarian cancer. *Lancet*.

[23] Horwitz S. B. (1994). Taxol (paclitaxel): mechanisms of action. *Annals of Oncology*.

[24] Bai T., Yokobori T., Altan B. (2017). High STMN1 level is associated with chemo-resistance and poor prognosis in gastric cancer patients. *British Journal of Cancer*.

[25] Lin X., Liao Y., Chen X., Long D., Yu T., Shen F. (2016). Regulation of oncoprotein 18/stathmin signaling by ERK concerns the resistance to taxol in nonsmall cell lung cancer cells. *Cancer Biotherapy & Radiopharmaceuticals*.

[26] Feng W., Xiaoyan X., Xuan Y. (2015). Silencing stathmin-modulating efficiency of chemotherapy for esophageal squamous cell cancer with paclitaxel. *Cancer Gene Therapy*.

[27] Burger R. A., Brady M. F., Bookman M. A. (2011). Incorporation of bevacizumab in the primary treatment of ovarian cancer. *The New England Journal of Medicine*.

[28] Perren T. J., Swart A. M., Pfisterer J. (2011). A phase 3 trial of bevacizumab in ovarian cancer. *The New England Journal of Medicine*.

[29] Lee E. K., Matulonis U. A. (2020). Emerging drugs for the treatment of ovarian cancer: a focused review of PARP inhibitors. *Expert Opinion on Emerging Drugs*.

[30] Poveda A., Floquet A., Ledermann J. A. (2021). Olaparib tablets as maintenance therapy in patients with platinum-sensitive relapsed ovarian cancer and a BRCA1/2 mutation (SOLO2/ENGOT-Ov21): a final analysis of a double-blind, randomised, placebo-controlled, phase 3 trial. *The Lancet Oncology*.

[31] Leiphrakpam P. D., Lazenby A. J., Smith L. M., Brattain M. G., Are C. (2021). Stathmin expression in metastatic colorectal cancer. *Journal of Surgical Oncology*.

[32] Kuang X. Y., Jiang H. S., Li K. (2016). The phosphorylation-specific association of STMN1 with GRP78 promotes breast cancer metastasis. *Cancer Letters*.

[33] Eggink L. L., Roby K. F., Cote R., Kenneth Hoober J. (2018). An innovative immunotherapeutic strategy for ovarian cancer: CLEC10A and glycomimetic peptides. *Journal for Immunotherapy of Cancer*.

[34] Bao P., Yokobori T., Altan B. (2017). High STMN1 expression is associated with cancer progression and chemo-resistance in lung squamous cell carcinoma. *Annals of Surgical Oncology*.

[35] Balachandran R., Welsh M. J., Day B. W. (2003). Altered levels and regulation of stathmin in paclitaxel-resistant ovarian cancer cells. *Oncogene*.

[36] Wang Z., He R., Xia H., Wei Y., Wu S. (2017). Knockdown of STMN1 enhances osteosarcoma cell chemosensitivity through inhibition of autophagy. *Oncology Letters*.

[37] Ren B., Cam H., Takahashi Y. (2002). E2F integrates cell cycle progression with DNA repair, replication, and G (2)/M checkpoints. *Genes & Development*.

[38] Chun J. N., Cho M., Park S., So I., Jeon J. H. (2020). The conflicting role of E2F1 in prostate cancer: a matter of cell context or interpretational flexibility?. *Biochimica Et Biophysica Acta. Reviews on Cancer*.

[39] Denechaud P. D., Fajas L., Giralt A. (2017). E2F1, a novel regulator of metabolism. *Frontiers in Endocrinology*.

[40] Ertosun M. G., Hapil F. Z., Osman N. O. (2016). E2F1 transcription factor and its impact on growth factor and cytokine signaling. *Cytokine & Growth Factor Reviews*.

[41] Chen Y. L., Uen Y. H., Li C. F. (2013). The E2F transcription factor 1 transactives stathmin 1 in hepatocellular carcinoma. *Annals of Surgical Oncology*.

[42] Hsu H. P., Li C. F., Lee S. W. (2014). Overexpression of stathmin 1 confers an independent prognostic indicator in nasopharyngeal carcinoma. *Tumour Biology*.

[43] Drucker E., Holzer K., Pusch S. (2019). Karyopherin alpha2-dependent import of E2F1 and TFDP1 maintains protumorigenic stathmin expression in liver cancer. *Cell Communication and Signaling*.

[44] Fan K., Zhang D., Li M. (2020). Carboxyl-terminal polypeptide fragment of MUC16 combing stathmin1 promotes gallbladder cancer cell migration and invasion. *Medical Oncology*.

[45] Ke B., Guo X. F., Li N. (2019). Clinical significance of stathmin1 expression and epithelial-mesenchymal transition in curatively resected gastric cancer. *Molecular and Clinical Oncology*.

[46] Chen Y., Zhang Q., Ding C., Zhang X., Qiu X., Zhang Z. (2017). Stathmin1 overexpression in hypopharyngeal squamous cell carcinoma: a new promoter in FaDu cell proliferation and migration. *International Journal of Oncology*.

[47] Wang J., Ni X., Shen S. (2021). Phosphorylation at Ser10 triggered p27 degradation and promoted gallbladder carcinoma cell migration and invasion by regulating stathmin1 under glucose deficiency. *Cellular Signalling*.

