# Supplementary material for: Overexpression of Stathmin 1 Predicts Poor Prognosis and Promotes Cancer Cell Proliferation and Migration in Ovarian Cancer
Source: Dis Markers. 2022 Feb 9;2022:3554100. doi: 10.1155/2022/3554100 (PMC8849943; doi:10.1155/2022/3554100)
Supplement: Supplementary Materials — Supplementary Figure 1: STMN1 was knocked down by siRNAs. (A, B) qRT-PCR was performed to measure STMN1 mRNA levels in SKOV3 and A2780 cells transfected with siRNAs. The sequences of STMN1 siRNA were as follows: S1, 5′-GCACGAGAAAGAAGUGCUU-3′; S2, 5′-CUGGAACGUUUGCGAGAGA-3′; and S3, 5′-GAACAACAACUUCAGUAAA-3′. [file 3554100.f1.zip › Supplementary table 1.pdf]

Gene Symbo	Gene ID	PCC
HMGN2	ENSG000001	0.69
TUBB	ENSG000001	0.68
MARCKSL1	ENSG000001	0.66
HMGN2P5	ENSG000002	0.65
GINS1	ENSG000001	0.63
UBE2T	ENSG000000	0.63
PRTFDC1	ENSG000000	0.61
SRSF12	ENSG000001	0.61
RFC4	ENSG000001	0.61
KIF15	ENSG000001	0.61
PCNA	ENSG000001	0.61
FBXO5	ENSG000001	0.61
AURKB	ENSG000001	0.61
MAD2L2	ENSG000001	0.61
GMNN	ENSG000001	0.61
PSMC3IP	ENSG000001	0.6
NCAPG	ENSG000001	0.6
DLGAP5	ENSG000001	0.6
SUV39H2	ENSG000001	0.6
SKP2	ENSG000001	0.6
SRSF7	ENSG000001	0.6
TMEFF1	ENSG000002	0.6
CENPA	ENSG000001	0.6
KLHL23	ENSG000002	0.6
CENPH	ENSG000001	0.6
TMSB15B	ENSG000001	0.6
DSN1	ENSG000001	0.59
RP11-722G7.	ENSG000002	0.59
CDC25A	ENSG000001	0.59
DNAJC8	ENSG000001	0.59
TMSB15A	ENSG000001	0.59
TIMELESS	ENSG000001	0.59
CENPO	ENSG000001	0.59
MSANTD3-TI	ENSG000002	0.59
EXO1	ENSG000001	0.59
TTK	ENSG000001	0.59
TPX2	ENSG000000	0.59
NCAPH	ENSG000001	0.58
CDC7	ENSG000000	0.58
FANCL	ENSG000001	0.58
CCNF	ENSG000001	0.58
PAFAH1B3	ENSG000000	0.58
HMGB2	ENSG000001	0.58
MCM2	ENSG000000	0.57
KIFC1	ENSG000002	0.57
CHEK1	ENSG000001	0.57
HDAC2	ENSG000001	0.57
AADAT	ENSG000001	0.57
ACTL6A	ENSG000001	0.57
USP39	ENSG000001	0.57
