# Supplementary figures and images for: Overexpression of Stathmin 1 Predicts Poor Prognosis and Promotes Cancer Cell Proliferation and Migration in Ovarian Cancer
Source: Dis Markers. 2022 Feb 9;2022:3554100. doi: 10.1155/2022/3554100 (PMC8849943; doi:10.1155/2022/3554100)

**A****SKOV3**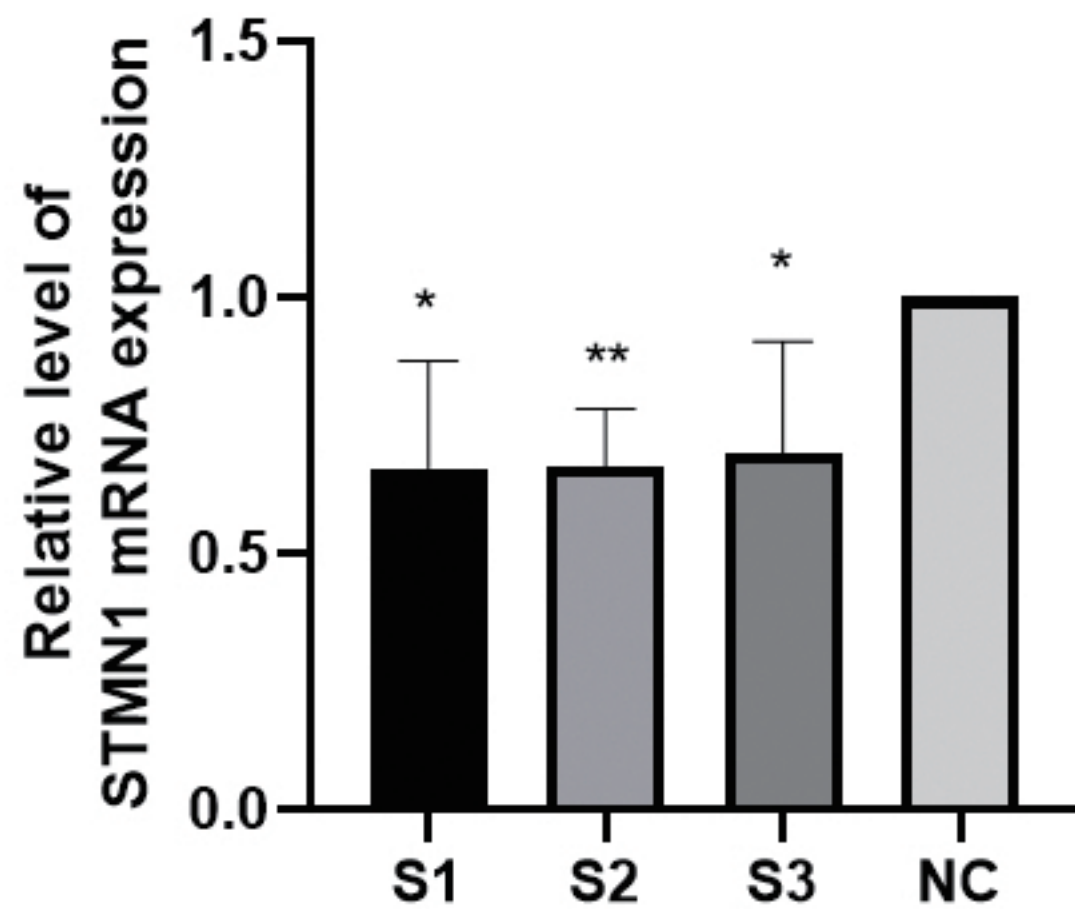**B****A2780**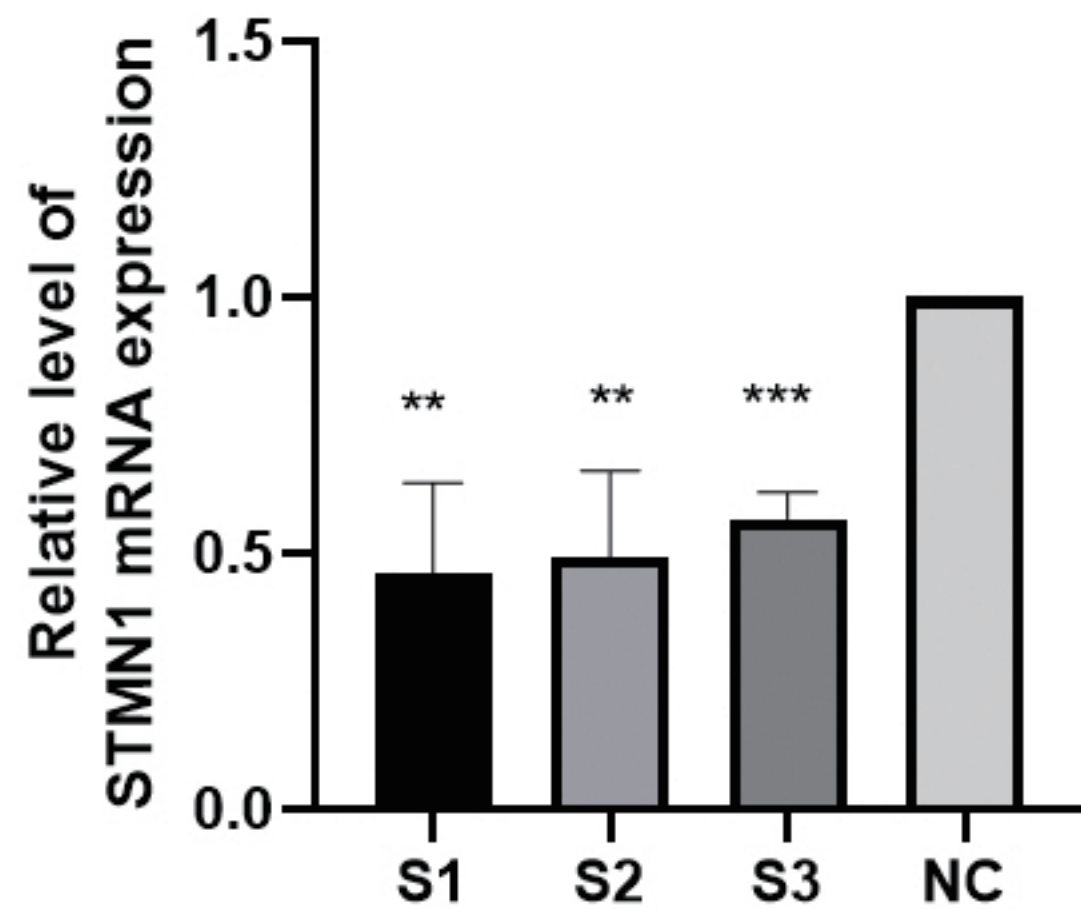

Supplement: Supplementary Materials — Supplementary Figure 1: STMN1 was knocked down by siRNAs. (A, B) qRT-PCR was performed to measure STMN1 mRNA levels in SKOV3 and A2780 cells transfected with siRNAs. The sequences of STMN1 siRNA were as follows: S1, 5′-GCACGAGAAAGAAGUGCUU-3′; S2, 5′-CUGGAACGUUUGCGAGAGA-3′; and S3, 5′-GAACAACAACUUCAGUAAA-3′. [file 3554100.f1.zip › supplementary fig 1.pdf]
